# Regulation of epithelial-mesenchymal transition through epigenetic and post-translational modifications

**DOI:** 10.1186/s12943-016-0502-x

**Published:** 2016-02-24

**Authors:** Silvia Juliana Serrano-Gomez, Mazvita Maziveyi, Suresh K. Alahari

**Affiliations:** Department of Biochemistry and Molecular Biology, LSUHSC School of Medicine, New Orleans, LA 70112 USA; Pontificia Universidad Javeriana, Bogota, Colombia

**Keywords:** Cancer, Metastasis, Epigenetic, Methylation, Therapy

## Abstract

The epithelial to mesenchymal transition (EMT) is a biological process in which a non-motile epithelial cell changes to a mesenchymal phenotype with invasive capacities. This phenomenon has been well documented in multiple biological processes including embryogenesis, fibrosis, tumor progression and metastasis. The hallmark of EMT is the loss of epithelial surface markers, most notably E-cadherin, and the acquisition of mesenchymal markers including vimentin and N-cadherin. The downregulation of E-cadherin during EMT can be mediated by its transcriptional repression through the binding of EMT transcription factors (EMT-TFs) such as SNAIL, SLUG and TWIST to E-boxes present in the E-cadherin promoter. Additionally, EMT-TFs can also cooperate with several enzymes to repress the expression of E-cadherin and regulate EMT at the epigenetic and post- translational level. In this review, we will focus on epigenetic and post- translational modifications that are important in EMT. In addition, we will provide an overview of the various therapeutic approaches currently being investigated to undermine EMT and hence, the metastatic progression of cancer as well.

## Background

Epithelial-mesenchymal transition (EMT) is a biological process in which non-motile, polarized epithelial cells undergo a series of biochemical alterations, becoming motile non-polarized mesenchymal cells with invasive capacity, resistance to apoptosis and altered biosynthesis of extracellular matrix (ECM) components. Epithelial and mesenchymal cells differ in their morphology and tissue organization. In a typical epithelium, cells are organized either as a single layer or in multi-layered sheets. In the latter case, structure is maintained through cell-cell interactions including tight junctions, gap junctions, cadherin based adherent junctions, desmosomes and ECM interactions [1, 2]. These junctions and interactions impede the movement of individual cells within the epithelial monolayer [3, 4]. Mesenchymal cells rarely establish tight junctions with surrounding cells and are embedded inside the extracellular matrix [5].

Cytoskeletal changes and cell signaling pathways are altered as a cell undergoes EMT. Processes known to contribute to EMT include the activation of transcription factors (TFs) such as SNAIL, SLUG and TWIST, altered expression of specific cell-surface proteins, reorganization and expression of cytoskeletal proteins, production of ECM-degrading enzymes, and changes in the expression of specific microRNAs [6, 7]. EMT is initiated following the dissolution of tight junctions resulting in the loss of apical-basal cell polarity [8, 9]. Other types of cell junctions are disassembled as well, such as gap and adherent junctions, leading to the loss of basement membrane integrity. The cytoskeleton also undergoes characteristic reorganization such as increased allocation of actin into stress fiber formation and the replacement of cytokeratin intermediate filaments by vimentin. These alterations enable the transition into a spindle-shaped cell morphology from a cuboidal/columnar precursor, correspond with an increased ability to invade surround tissue [10–12]. A cell is considered to have undergone EMT following the loss of epithelial marker expression in tandem with the development of mesenchymal marker expression. Key epithelial markers lost include E-cadherin (*CHD1*), Mucin-1, Cytokeratins (such as CK19, CK18, CK8), Occludin and Desmoplakin. Oppositely, markers gained during the process include N-cadherin, Vimentin, Smooth Muscle Actin (αSMA), Fibronectin, and Vitronectin, which together comprise the key mesenchymal markers [6, 13–16]. In addition numerous proteins not located on the cell surface also undergo key changes in localization. β-catenin, a component of the cadherin complex is one such example. During EMT, β-catenin dissociates from the cadherin complex and is translocated into the nucleus where it behaves as a transcription factor, regulating the expression of several genes in key pathways such as Wnt signaling. Importantly, the changes observed in cells to revert back to a epithelial-like phenotype upon arrival at a suitable location to colonize, a process prudently entitled mesenchymal to epithelial transition (MET) [5].

EMT has been classified into three categories: type I, type II and type III [17, 18]. Type I occurs during embryogenesis where cells need to migrate to adjacent tissues in order to form new organs and tissues [5]. Type II is associated with the wound healing, whereby fibroblasts repair or rebuild tissues [6]. Unlike types I and II which perform necessary physiologic functions, type III is a pathophysiologic adaptation of the process, and is closely associated with progression of neoplasia occurring in cells containing certain epigenetic and genetic changes [4, 19]. It is currently theorized that exploitation of the normal EMT signaling pathways provides the molecular genetic basis for how neoplastic (but differentiated) cells can shed their epithelial characteristics and acquire migratory properties. Having undergone such a change, the cells are subsequently able to invade tissues surrounding the primary tumor, extravasate into lymphatics or blood vessels, travel to distant sites through the circulation, and ultimately colonize a metastatic niche [18, 20]. It is important to highlight that oncogenic EMT is a transient process that may function in a paracrine fashion and is followed by MET once the tumor cells reach the metastatic site [21].

The EMT program is activated by multiple signaling pathways as well as several epigenetic and post-translational modifications such as methylation, acetylation, phosphorylation, glycosylation, hydroxylation and SUMOylation. Epigenetic modifications including modification of histone protein tails, and DNA promoter regions, play a key role in regulating gene expression by defining whether chromatin at a given genomic locus will be transcriptionally active or inactive [22]. Post translational modifications are covalent modifications that occur after transcript has been translated into protein [23]. Improving our understanding of how these modifications function in the regulation of EMT is of crucial importance, and likely instance where novel therapeutics might be developed to better treat diseases such as cancer [24]. Since the EMT program is regulated dually by epigenetic and post translational modifications, we will focus closely on these two mechanisms as they pertain to EMT in this review. In addition, we will provide a current overview of the various therapeutic approaches currently being investigated to undermine EMT.

## E-cadherin as a key epithelial marker

The *CDH1* gene is located on chromosome 16q22.1 and codes for the subtype of cadherin protein expressed by epithelial cells (E-cadherin). Functionally, E-cadherin behaves as a tumor suppressor gene and plays diverse roles in regulating cell polarity, differentiation, migration and stem cell-like properties. In the context of cell polarity, E-cadherin binds to adjacent cells creating an intercellular complex that forms epithelial barriers. The extracellular portion of E-cadherin binds to cadherins on an adjacent cell creating a bridge between the cytoskeletons of contiguous cells. The intracellular domain of E-cadherin interacts with β-catenin, which itself is linked actin filaments within the cells via a linker protein called α-catenin [25–27].

Down-regulation or inactivation of *CDH1* has been frequently observed during tumor cell progression, and several mechanisms have been proposed [28]. These include germline mutations [29, 30], promoter hypermethylation [31, 32] and upregulation of E-cadherin transcriptional repressors [10], alternatively known as EMT transcription factors (EMT-TFs). Transcription factors such as SNAIL, SLUG, ZEB1, and ZEB2/SIP1 are considered direct repressors of E-cadherin as they bind to E-boxes present on the *CDH1* promoter [10, 33, 34]. Indirect repressors include bHLH proteins (TWIST1 and TWIST2), homeobox proteins (GSC and SIX1), the bHLH factor E2.2 and the forkhead-box protein FOXC2 [2, 10]. Additionally, while the TWIST proteins are commonly recognized as an indirect repressors of *CDH1*, they can also bind directly on E-boxes 2 and 3 present on the *CDH1* promoter to repress its expression [35].

## Epigenetic modifications during EMT

Epigenetic modifications allow for regulation of gene activity and expression without altering the DNA sequence. Such changes include methylation of cytosine residues in CpG dinucleotides in the DNA; and histone modifications at N-terminal tails including acetylation, methylation, phosphorylation and ubiquitination [36, 37]. DNA methylation is a well-studied epigenetic event associated with transcriptional silencing resulting from disrupted binding affinity between gene promoters and their cognate TFs. Modification of histones on the other hand alters gene expression by reshaping the anatomy of the nearby chromatin resulting in alterations in the ability of transcriptional machinery to access genes within the region [36]. Below, we will discuss each of these epigenetic modifications in further detail.

### DNA methylation

DNA methylation is one of the fundamental epigenetic modifications in mammals. It occurs at the 5-position of cytosine (5mC) in CpG dinucleotides and is catalyzed by DNA methyltransferases (DNMTs) [38, 39]. The DNMT family is composed of four members: DNMT1, DNMT3A, DNMT3B, and DNMT3L. DNMT1 maintains DNA methylation during DNA replication, while DNMT3a and DNMT3b regulate *de novo* DNA methylation primarily during embryonic development. The inactivation of *CDH1* by hypermethylation is a common event in multiple human carcinomas including breast, bladder, lung, liver, gastric and prostate [32, 40–42]. Additionally, promoter hypermethylation of the *CDH1* gene is positively associated with EMT in breast cancer cell lines, corresponding with the increased potential for invasion and metastasis observed in these cells [43].

In murine cells, oncogenic Ras has been shown to induce EMT in cooperation with certain serum factors [44]. Dumont and colleagues [45] worked with a model of immortalized Human Mammary Epithelial cells (HMEC) with repressed p16INK4A (vHMEC)-expressing oncogenic Ras (vHMEC-ras) and showed that these cells change morphology and became motile when cultured in serum-rich media. Moreover, they reported changes in the methylation status of several genes including *CDH1* promoter as well as *ESR1* (which codes for estrogen receptor) and TWIST in cells with a mesenchymal phenotype that were exposed to 10 % serum but not in cells with the epithelial phenotype.

**Fig. 1:**
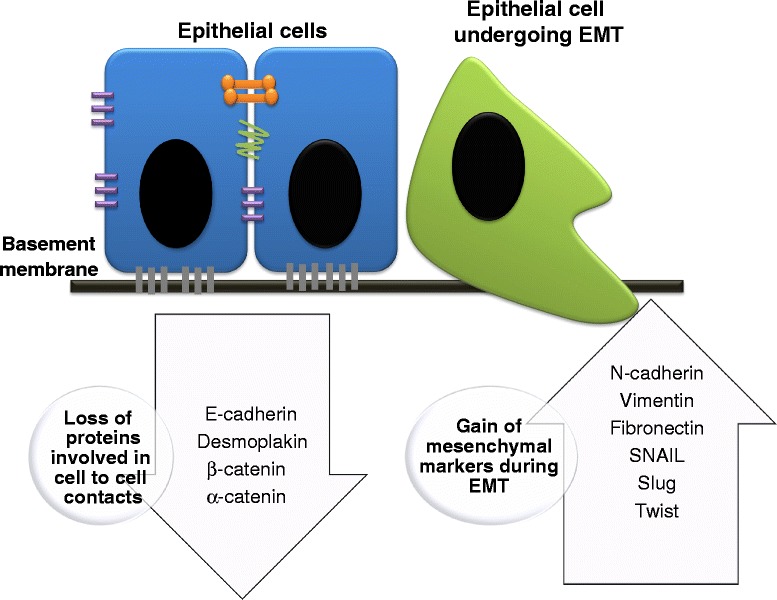
Changes in the molecular markers during EMT. E-cadherin, Desmoplakin, β-catenin and α-catenin are cell-cell contact proteins that are lost during EMT. N-cadherin, Vimentin, Fibronectin, SNAIL, Slug and Twist are mesenchymal markers that are gained during EMT

Several transcriptional factors including ZEB1, SNAIL and TWIST regulate *CDH1* expression (Fig. 1). ZEB1 is a transcription factor that plays important roles in embryogenesis and cell differentiation [46]. ZEB1 represses *CDH1* transcription by its binding to two E-box sequences in the promoter. ZEB1 can also regulate *CDH1* expression at the epigenetic level. Basal-like breast cancer (BLBC) is a breast cancer subtype enriched with expression of mesenchymal genes and reduced expression of epithelial genes including E-cadherin [47]. Downregulation of *CDH1* in BLBC is mediated by ZEB1, which recruits DNMT1 to the *CDH1* promoter to maintain the methylation status in the promoter [38]. These results suggest that ZEB1 acts as a transcriptional repressor and an epigenetic modulator to induce EMT in breast cancer. Although the hypermethylation of *CDH1* has been well-associated with EMT, McDonald and colleagues showed that the DNA methylation was unchanged during EMT in a model of mouse hepatocytes treated with TGF-β [48]. It is important to note that the mechanisms leading to EMT could be different in normal compared to cancer cells. In fact, Dumont and colleagues [45] suggested that TGF-β signaling and oncogenic stress induce EMT in human cells.

DNA methylation inhibitors such as 5-aza-2′-deoxycytidine (5-aza-CdR) have been found to function as anticancer agents in light of their ability to reactivate tumor suppressor gene expression [49]. However, Ateeq and colleagues [50] have hypothesized that such demethylating agents might also increase the risk of tumor metastasis by inadvertently activating genes involved in invasion and metastasis. They tested the DNA methylation in a set of genes involved in metastasis, angiogenesis and cell cycle regulation as well as in the tumor suppressor gene *RASSF1A* after treatment with 5-aza-CdR in noninvasive human breast cancer cells MCF7 and ZR-75-1. They found that although treatment with 5-aza-CdR increased the expression level of *RASSF1A,* it also increased the expression of genes involved in metastasis, such as *uPA, SNCG*, and *CXCR4* [50, 51]. The mechanism responsible for the increase in the expression of those genes was attributed to demethylation at their regulatory regions. Such findings highlight the fundamental importance of DNA methylation in contribution to the activation of pro-metastatic genes, and consequently, EMT.

### Histone modifications

#### Methylation

SNAIL is a zinc finger transcription factor that regulates EMT during development of the mesoderm and neural crest [52]. In breast cancer, the expression of *SNAI1* has been shown to be upregulated in recurrent tumors and in addition, is associated with metastasis and decreased relapse-free survival [52]. Similar to ZEB1, SNAIL suppresses the transcription of *CDH1* by binding to E-boxes in the *CDH1* promoter [53]. It can also cooperate with histone methyltransferases (HMT) and DNMTs to modulate the expression of *CDH1* (Table 1) [37].

**Table 1:** Key Histone Methyltransferases in EMT

HMT	Histone	Promoter
KDM6B	H3K27m3	*SNAI1*
MMSET	H3K36m2	*TWIST*
LSD1	H3K4m2	*CDH1*
Suv39H1	H3K9m3	*CDH1*
SET8	H4K20m1	*CDH1*
		C*DH2*
G9a	H3K9m1/2	*CDH1*

KDM6B demethylates H3K27 at the *SNAI1* promoter. MMSET can di- or trimethylate H3K36 at the *TWIST* promoter. LSD1 methylates H3K4m2 on *CDH1*. Suv39H1 can trimethylate H3K9 on the *CDH1* promoter. SET8 methylates H4K20 on the *CDH1* and *CDH2* promoters. G9a is responsible for mono and dimethylation of H3K9

##### KDM6B in EMT:

 KDM6B (also known as JMJD3) is an α-ketoglutarate dependent demethylase containing a conserved Jumonji C (JmjC) domain. This enzyme is responsible for the demethylation of di- and trimethyl-lysine 27 (H3K27m2/3) on histone H3 (Table 1). H3K27m2/3 is an epigenetic modification associated with gene silencing [54, 55]. It has been reported that KDM6B expression is higher in metastatic prostate cancer [56]. It is also highly expressed in invasive breast carcinomas compared to normal tissues. Moreover, during TGFβ-induced EMT, TGF-β activates KDM6B which then demethylates H3K27m3 at *SNAI1* promoter. This epigenetic modification activates the transcription of *SNAI1*. This suggests a role for KDM6B during tumor invasion which was demonstrated in MDA-MB-231 cells [57].

##### MMSET in EMT:

TWIST is a transcription factor that regulates cell migration and tissue reorganization during early embryogenesis but also plays an important role in EMT and tumor metastasis [58, 59]. TWIST induces the down-regulation of E-cadherin and increased expression of mesenchymal markers such as Fibronectin, Vimentin, αSMA, and N-cadherin [60]. The overexpression of TWIST has been associated with poor prognosis in human cancers [58, 60, 61]. One mechanism by which TWIST induces EMT is through its interaction with a HMT. MMSET (also known as WHSC1 or NSD2) is a HMT that was first identified as a candidate gene for Wolf–Hirschhorn Syndrome (WHS). MMSET can di- or trimethylate histone H3 at lysine 36 (H3K36) (Table 1) [25, 62]. In solid tumors, MMSET is overexpressed and associated with poor prognosis [63]. MMSET binds to the *TWIST* promoter and increases its methylation at H3K36m2. This change results in *TWIST* activation, which contributes to prostate cancer progression.

##### LSD1 in EMT:

 Several histone demethylases regulate E-cadherin expression. The histone demethylase LSD1 (also known as KDM1A and AOF2) plays an important role in EMT [64]. It has been shown that SNAIL interacts with LSD1 through its SNAG (SNAI1/GF) domain and recruits LSD1 to *CDH1* promoter. As a consequence, methyl groups on lysine 4 of histone H3 will be removed (H3K4m2) (Table 1). The demethylation suppresses the expression of *CDH1* and enhances cell invasion. This mechanism suggests that LSD1 could be a good target for a new therapeutic modality. Another proposed therapeutic approach could be to construct peptides that mimic the structure of the SNAG domain of SNAIL to inhibit its function during EMT and cancer progression [64, 65].

##### SUV39H1 in EMT:

Suv39H1 (suppressor of variegation 3–9 homolog 1) is a histone methyltransferase responsible for the trimethylation of histone H3 at lysine K9 (H3K9m3) in the *CDH1* promoter (Table 1). The mechanism responsible for this post transcriptional modification is through the interaction of SNAIL with Suv39H1. This interaction leads to the recruitment of Suv39H1 to the *CDH1* promoter to repress its transcription. The suppression of Suv39H1 could be a good therapeutic approach to rescue *CDH1* expression in cancers where the DNA methylation levels are high, such as in BLBC [66].

##### SET8 in EMT:

SET8 (also known as PR-Set7/9, SETD8 or KMT5A) is a HMT of the SET domain-containing methyltransferase family. SET8 methylates lysine 20 of histone H4 (H4K20), and regulates transcription both positively as well as negatively (Table 1) [67]. SET8 mediates the transcriptional activation of *WNT* target genes [59, 68] and regulates the S-phase progression of the cell cycle [69]. In EMT, SET8 has dual functions; activation of N-cadherin and repression of *CDH1* expression. The physical interaction between SET8 and TWIST promotes the recruitment of SET8 to the *N-cadherin* promoter and induces H4K20 methylation (H4K20m1). This modification results in activation of *N-cadherin* expression. In contrast, H4K20m1 methylation of the *CDH1* promoter represses *CDH1* expression suggesting that methylation has two distinct functions.

##### G9A in EMT:

 G9a is a HMT responsible for mono and dimethylation of histone H3 at lysine K9 (H3K9m1 and H3K9m2) (Table 1). Methylation at this residue is associated with repression of transcription [37]. It has been shown that the c-terminal domain of SNAIL interacts with ankyrin repeat and SET domains of G9a, and recruits G9a and DNMTs (DNMT1, DNMT3a and DNMT3b) to the *CDH1* promoter resulting in its methylation. The knockout of G9a restored *CDH1* expression and results in the inhibition of cell migration and invasion in a model of breast cancer [70]. Although DNMT1 can modulate the expression of *CDH1* through increasing promoter methylation levels, it could govern expression of *CDH1* in a methylation-independent manner as well. DNMT1 can interact with SNAIL to prevent its interaction with the *CDH1* promoter leading to full expression of *CDH1* [71].

When considered together, the examples discussed above provide the current framework for the epigenetic regulation of EMT-TFs. This type of regulation is responsible for the key TFs which lead to the suppression of E-cadherin- a fundamental step in the initiation of EMT program and thus is of paramount importance to cells undergoing EMT. Designing drugs to target the components of this process is a budding field of cancer pharmacotherapy, but is complicated by the variable effects which histone methylation can have on gene expression (e.g., methylation at H3K36m2 activates *TWIST* gene expression, while methylation at H3K27 inactivates *SNAIL* gene expression). Thus, future treatments will need to get target specific methyltransferase or demethyltransferases in order to be effective, rather than simply altering histone methylation in a unilateral fashion.

In the context of EMT, hypermethylation of E-cadherin is an epigenetic modification associated with the invasive capacity of cancer cells and occurs through the interactions of EMT-TF with several HMTs and DNMTs. The interactions among these complexes can either repress the expression of *CDH1*, inducing a mesenchymal state, or repress the expression of the EMT-TF, thereby maintaining an epithelial state. Currently, several DNA methylation inhibitors are approved by the US Food and Drug Administration (FDA). One such inhibitor, 5-Azacytidine, is well known to have good efficacy in treating myelodysplastic syndromes [72, 73]. The identification of these epigenetic markers within a tumor would be helpful to develop new therapeutic approaches by targeting enzymes that modulate EMT in cancers.

#### Acetylation

Histone acetylation and deacetylation play important roles in the regulation of transcription [74]. Histone acetylation is catalyzed by Histone Acetyl Transferases (HATs), an event which is usually associated with transcriptionally active chromatin. As a result of acetylation, positively charged lysines are neutralized which increases accessibility to DNA. On the other hand, histone deacetylases (HDACs) catalyze the removal of the acetyl residues in the chromatin of inactive regions [31, 74, 75]. HATs and HDACs are usually part of multi-protein complexes composed of co-activators or co-repressors that are recruited to specific DNA sequences to determine the acetylation status [31]. Multiple HDACs have been identified in mammals and are grouped according to their homology to deacetylases in yeast. HDAC1 and HDAC2 are class I enzymes and have been found to be overexpressed in some cancers [76]. HDAC1 is highly expressed in hepatocellular carcinoma, breast, and liver, prostate, gastric and colon cancer; and HDAC2 is overexpressed in colorectal, cervical and gastric cancer [77, 78].

Histone deacetylation is at least one mechanism by which the aforementioned SNAIL represses E-cadherin expression. SNAIL directly interacts with E-cadherin promoter and recruits HDAC1, HDAC2 and the co-repressor Sin3A to the *CDH1* promoter to silence its expression by deacetylation of histones H3 and H4. This effect was abolished by treatment with the HDAC inhibitor Trichostatin A (TSA) [31]. HDAC1 is also required for TGFβ1-induced EMT in hepatocytes and frequently overexpressed in hepatocellular carcinoma (HCC), suggesting a strong connection between HDAC1 and the invasive properties of HCC. HDAC1 has been shown to repress the epithelial genes such as *CDH1* and *ZO-1* [79]. In pancreatic cancer, downregulation of E-cadherin occurs through ZEB1, which recruits HDAC1 and HDAC2 to the *CDH1* promoter to silence its expression [80]. In contrast, in ZEB1 knockdown cells, HDACs cannot be recruited, and as a consequence, E-cadherin expression is induced in such cases, implicating a significant role of HDAC in EMT.

Also, E-cadherin expression is regulated by SMAR1 (Scaffold/Matrix attachment region-binding protein) which forms a complex with HDAC1 and binds to the *SLUG* promoter. This results in the repression of the transcription of *SLUG*, and thus restores E-cadherin expression [81]. As described above, TWIST is considered an indirect repressor of E-cadherin. The Mi2/nucleosome remodeling and deacetylase (Mi2/NuRD) complex contains multiple proteins that have activities similar to histone deacetylase and chromatin-remodeling ATPase. TWIST can interact with proteins in the Mi 2/NuRD/MTA2 complex and recruit this complex to the *CDH1* promoter that results in the repression of E-cadherin expression [82].

Another family of proteins that regulate acetylation is the sirtuins. Sirtuins are class III histone deacetylases that use nicotinamide adenine dinucleotide (NAD+) to mediate the deacetylation of histone and non-histone substrates. Thus, sirtuin activity is regulated by the intercellular [NAD+]/[NADH] ratio [74, 83]. The sirtuin family of proteins is composed of seven members (SIRT1-7). SIRT1, 2, 3 and 5 target proteins in the nucleus, cytoplasm and mitochondria for acetylation, while SIRT 4 and 6 regulate ADP ribosylation [84]. SIRT1 deacetylates histone H4 lysine 16 (H4K16) as well as histone H3 lysine 9 (H3K9) and histone H3 at lysine 14 (H3K14). Some reports indicate that SIRT1 may provide genetic stability and suppress tumor formation [85]. In contrast, other studies indicate that SIRT1 levels are high in cancer samples and this is associated with poor prognosis and metastasis [86–88]. Thus the exact role of SIRT1 in cancer is very controversial. An example of how SIRT1 acts as a negative regulator of EMT is in oral squamous cell carcinoma (OSCC). The expression of SIRT1 in OSCC decreases TGF-β-mediated cell migration and metastasis. This occurs due to high levels of SIRT1 expression and leads to the deacetylation of SMAD4, a downstream target of TGF-β which suppresses the expression of MMP7. Downregulation of SIRT1 leads to SMAD4 hyperacetylation and MMP7 hyperactivation that results in the degradation of E-cadherin, the release of β-catenin from cell junctions and translocation to the nucleus to promote metastasis in OSCC cells [89]. On the other hand, in prostate cancer, SIRT1 induces EMT [90]. The mechanism is through ZEB1, which recruits SIRT1 to the *CDH1* promoter to deacetylate histone H3. As a consequence, the RNA polymerase binding and *CDH1* transcription are reduced. These results suggest that SIRT1 may be a good therapeutic target for prostate cancer among others [91].

#### Interplay between HDAC and HMT to regulate EMT

Hypoxia is a microenvironmental condition known to promote tumor progression through the stabilization of Hypoxia Inducible Factor-1 (HIF-1). HIF-1α activates genes involved in cellular processes such as angiogenesis, invasion and EMT [24, 58]. Under hypoxic condition, different chromatin modifiers regulate EMT [92]. WDR5 is a WD40 repeat protein and a HMT that is essential for histone H3 lysine 4 (H3K4) methylation [92, 93]. HIF-1α activates the expression of HDAC3 that deacetylates H3K4 on both mesenchymal genes (Vimentin and N-cadherin) and epithelial genes (E-cadherin and Plakoglobin). To modulate EMT, HDAC3 additionally recruits the WDR5/HMT complex to mesenchymal promoters and increases the methylation levels of H3K4m2 to promote gene activation and EMT. The knockdown of WDR5 abolishes the activation of mesenchymal genes during hypoxia [92]. These results suggest that hypoxia-induced EMT is regulated by interplay between histone deacetylases (HDAC3) and histone methyltransferases complexes.

HDACs mediate epigenetic mechanisms with important roles in cell cycle regulation, cell proliferation and differentiation [94]. The activity of HDACs has also been associated with the development and progression of fibrotic disorders [95], as well as cancer, and thus it is important to explore potential applications of HDAC inhibitors to inhibit EMT. However, the role of certain HDACs is still controversial, which hinders the development of a viable therapeutic option [81]. However, therapeutic use of HDAC inhibitors such as SAHA (suberoylanilide hydroxamid acid), valproic acid (VPA) and trichostatin A (TSA) has been tested in several models of cancer showing promising results [96]. For example, in triple negative breast cancer, Panobinostat an inhibitor of HDAC class I, II and IV, inhibits proliferation as well as increases the expression of E-cadherin [97]. In summary, complete understanding of histone acetylation in EMT will lead to more effective cancer treatments.

## Post-translational modifications during EMT

Post- translational modifications (PTMs) are covalent modifications that occur after RNA is translated into protein [23]. These covalent modifications include the addition of a modifying chemical group or the addition of another small protein to one or more residues of the target protein [98]. PTM can either occur on a single residue within the protein or on multiple residues undergoing the same or different modification [99]. In this review we will focus on hydroxylation, phosphorylation, SUMOylation and glycosylation (Table 2).

**Table 2:** Post - Translational Events in EMT.

Post- translational event	Modified protein
Hydoxylation	HIF1A
Phosphorylation	SNAIL
	Par6
SUMOylation	FoxM1
	TFAP2C
	SIP1
Glycosylation	SNAIL

Hydroxylated HIF-1α is stabilized and promotes EMT by decreasing epithelial markers such as E-cadherin and gaining mesenchymal markers such as α-SMA and FSP1. Phosphorylation of SNAIL mediated either by PKD1 or GSK-3β results in SNAIL degradation by the proteasome. Phosphorylated Par6 interacts with the E3-ubiquitin ligase Smurf-1 that targets RhoA for degradation, leading to the disassembly of tight junctions. FoxM1 SUMOylation leads to repression of miR-200 tumor suppressors that enhance the expression of E-cadherin and suppress the expression of ZEB1 and ZEB2. TFAP2C undergoes SUMOylation that blocks its ability to induce the expression of luminal genes and helps it to maintain basal like features. SIP1 SUMOylation leads to the recruitment of the co-repressor CtBP which maintains *CDH1* expression. Lastly, SNAIL is glycosylated under hyperglycemic conditions to promote EMT

### Hydroxylation

Hydroxylation is a post-translational modification that occurs in proline residues [100]. The HIF-prolyl hydroxylases (HPHs) also known as prolyl hydroxylase domain (PHD) proteins are enzymes that use oxygen and 2-oxoglutarate (2-OG) as co-substrates, and iron and ascorbate as cofactors. PHDs sense the cytosolic concentration of oxygen and as a result they regulate HIF-1α. PDHs hydroxylate HIF-1α at two proline residues (Pro402 and Pro564) located in the oxygen-dependent degradation domain (ODDD) [100].

**Fig. 2:**
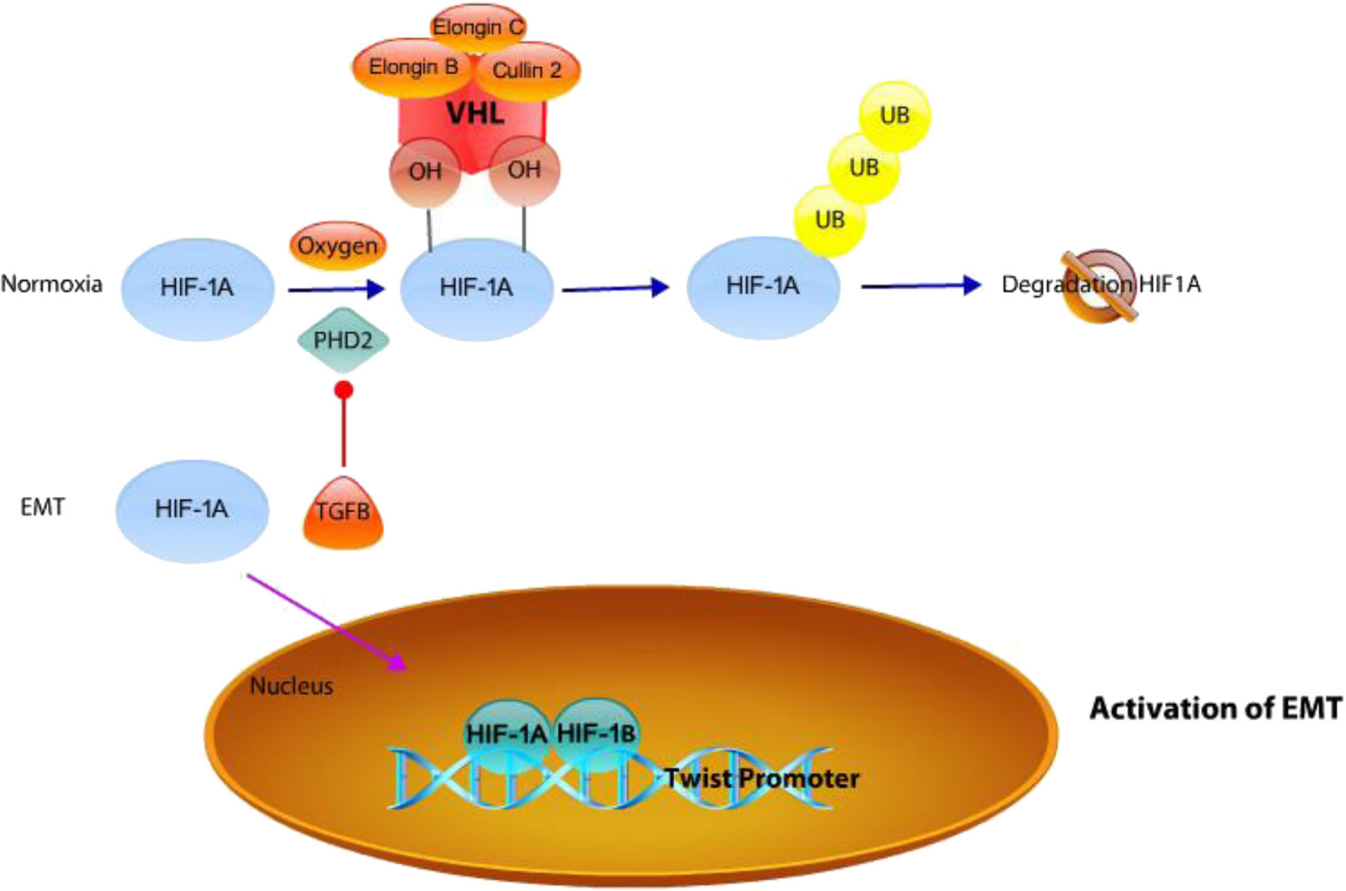
Hydroxylation of HIF1A. In the presence of oxygen, HIF-1α is hydroxylated by PHD2. This hydroxylation is recognized by a protein complex containing Cullin 2, VHL, Elongin B, and Elongin C that targets HIF-1α for ubiquitination and leads to proteasome degradation of HIF1α. During EMT, the activation of TGF β decreases transcription of PHD2, which leads to the stabilization of HIF-1α. The stabilized HIF-1α binds to the hypoxia-response element (HRE) of the TWIST promoter along with HIF-1β to induce its transcription. (*Blue arrowhead* indicates sequential patterns.  indicates inhibition. *Purple arrowhead* indicates translocation.)

HIF-1α activates genes involved in cellular processes such as angiogenesis, invasion and EMT [24]. Under normoxia, HIF-1α is hydroxylated by a family of oxygen dependent prolyl hydroxylases (PHD1–3). The HIF-1α-associated prolyl hydroxylase PHD2 is an important cellular oxygen sensor that regulates HIF-1α degradation under normoxia [101]. The hydroxylated HIF-1α is recognized by a protein complex consists of the tumor suppressor Von Hippel-Landau protein, Elongin B, Elongin C, and Cullin 2 and possesses E3 ubiquitin ligase activity, which targets HIF-1α for polyubiquitination and degradation. In contrast, under hypoxic conditions, prolyl hydroxylation is inhibited leading to HIF-1α stabilization [101]. There are elements in the tumor microenvironment such as TGFβ that influence the activity of HIF-1α under normoxia. TGFβ decreases PHD2 mRNA and protein levels. As a consequence, hydroxylation of the ODDD domain of HIF-1α goes down, which results in an increase of HIF-1 stability [101] (Fig. 2). TGFβ decreases PHD2 expression in a SMAD2/3 dependent manner. As a result of PHD2 decreased expression, HIF-1α is stabilized and promotes EMT by decreasing epithelial markers such as E-cadherin and gaining mesenchymal markers such as α-SMA and Fibroblast Specific Protein 1 (FSP-1). TWIST is a downstream target of HIF-1 α and has a hypoxia-response element (HRE) at the promoter region. Once HIF-1 α is stabilized, it binds to the HRE of TWIST and promotes EMT. This suggests that one of the mechanisms by which hypoxia induces EMT is through the direct activation of TWIST (Fig. 2).

### Phosphorylation

Protein phosphorylation is the most common post translational modification and is essential for the regulation of multiple molecular pathways involved in processes such as metabolism, transcription, differentiation and apoptosis. Phosphorylation is catalyzed by enzymes called Protein Kinases (PK) that catalyze the transfer of γ-phosphate of ATP to serine, threonine or tyrosine residues on target proteins. Protein Phosphatases (PP) catalyze the reverse process [102–104].

**Fig. 3:**
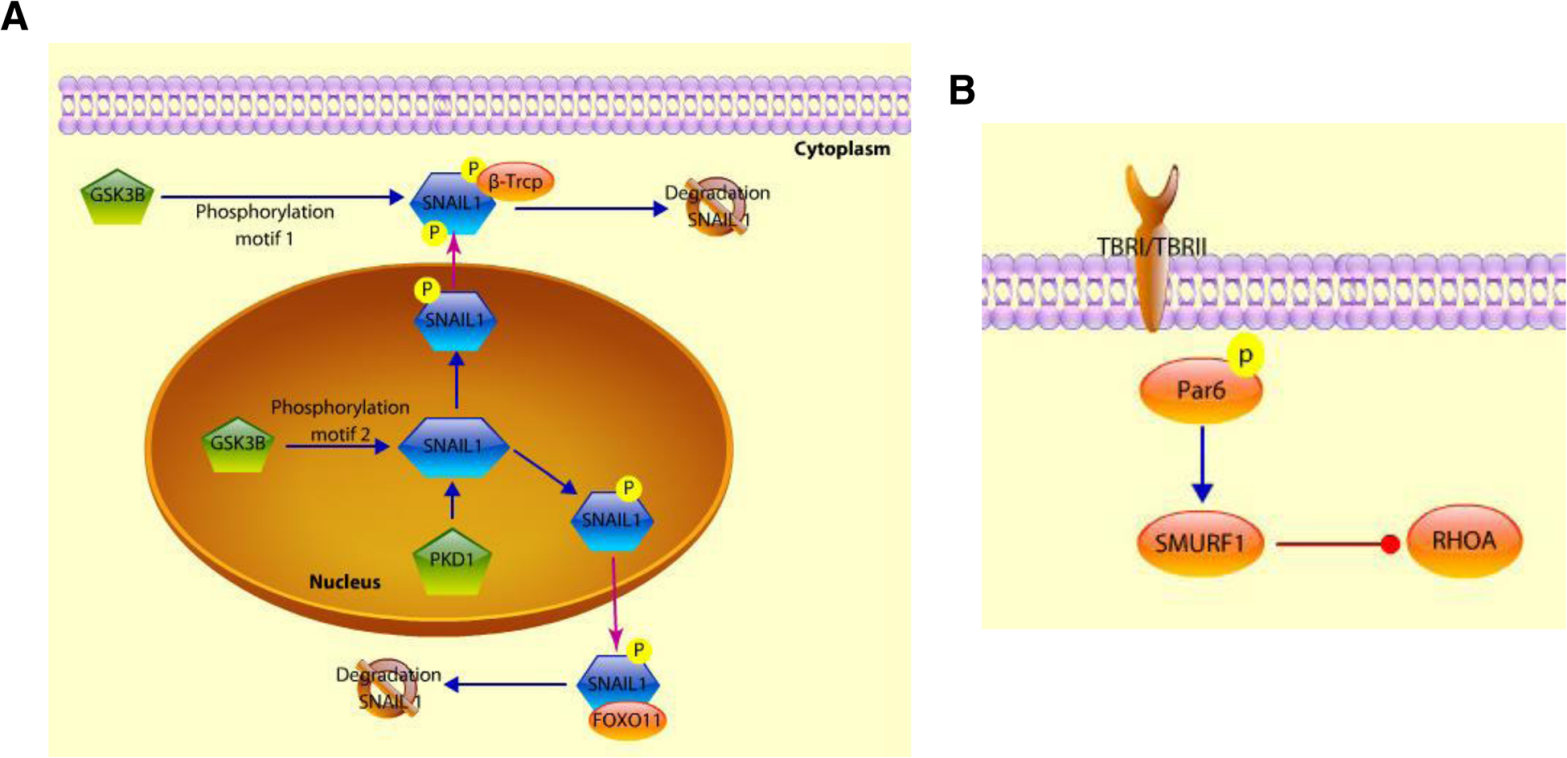
Phosphorylation of SNAIL and Par6. (*Blue arrowhead* indicates sequential patterns.  indicates inhibition. *Purple arrowhead* indicates translocation.) **a**. SNAIL phosphorylation that suppresses EMT. GSK-3β phosphorylates SNAIL at two consecutive motifs. First, the phosphorylation at the second motif induces the cytoplasmic translocation of SNAIL from the nucleus. In the cytoplasm, SNAIL is phosphorylated on motif 1, and this phosphorylation is recognized by β-Trcp which targets it for proteosomal degradation of SNAIL. PKD1 is another kinase that phosphorylates SNAIL so it can be recognized by β-Trcp and FOXO11 that target it for proteosomal degradation. **b**. Mechanisms of EMT activation mediated by TGFβR. The activation of TGFβR results in the phosphorylation of Par6, and in turn activation of SMURF1 that targets RHOA degradation by the proteasome, which contributes to the disassembly of the tight junctions

Phosphorylation is another post- translational modification known to control SNAIL [105]. GSK-3β, a well-known kinase involved in many signaling pathways, phosphorylates SNAIL at two consecutive motifs that control its ubiquitination and subcellular localization. Firstly, GSK-3β binds to SNAIL and phosphorylates SNAIL at motif 2, which induces the nuclear export of SNAIL. Later, the phosphorylation at motif 1 promotes the ubiquitin-mediated proteasome degradation of SNAIL by β-Trcp. The inhibition of GSK-3β results in the upregulation of SNAIL and downregulation of E-cadherin that results in the activation of the EMT program [106]. SNAIL activity can also be regulated by phosphorylation on Ser11. This residue is located within the SNAG domain that corresponds to the SNAIL transcriptional repression domain. In prostate cancer, the protein kinase D1 (PKD1) acts as a regulator of EMT. PKD1 mediates the phosphorylation at Ser11 of SNAIL. Once phosphorylated, 14-3-3σ binds to SNAIL and SNAIL can no longer function on E-cadherin [107]. These results suggest that PKD1 acts as a tumor and metastasis suppressor as it regulates Snail-mediated EMT. Furthermore, phosphorylation at Ser 11 of SNAIL mediated by PKD1 serves as a binding site for FBXO11, an E3 ligase that promotes SNAIL ubiquitination and degradation [108]. These results establish a mechanism of post translational regulation of EMT mediated by the PKD1-FBXO11-SNAIL axis (Fig. 3a).

Several tyrosine kinase receptors can also activate EMT. Epidermal Growth Factor Receptor (EGFR) is a transmembrane glycoprotein with an intracellular protein tyrosine kinase domain. The activation of EGFR by EGF, results in the activation of multiple pathways including ERK-1/2, PI3K and Stat3 via phosphorylation [109]. The overexpression of EGFR is associated with tumor progression and poor prognosis [110]. Lu and colleagues showed that the treatment of cancer cells that overexpress EGFR with EGF increased cell motility and invasion by the rapid dephosphorylation of focal adhesion kinase (FAK). Tumor cells with inactive FAK are less adherent to the extracellular matrix (ECM), which promotes cell motility, invasion, and metastasis [110]. The disassembly of tight junctions during EMT can occur in a SMAD independent manner as well. TGFβRI and Par6 coexist in the tight junctions. Upon stimulation of TGFβ, the TβRI- TβRII hetero-dimerize, resulting in a complex containing TβRII/TβRI and Par6 in each tight junction. This interaction results in phosphorylation of Par6 at Ser345, which is mediated by TβRII [111]. Phosphorylated Par6 interacts with the E3-ubiquitin ligase Smurf-1 that targets RhoA for degradation, leading to the disassembly of tight junctions (Fig. 3b). Thus TGF- beta signaling has good therapeutic value [112, 113].

Although aberrant phosphorylation of multiple proteins in different pathways is a major event during cancer development and progression, the design of viable therapeutic targets that inhibit this event seems challenging and the consequences may not be beneficial in all carcinomas. For example, phosphorylation of SNAIL mediated either by PKD1 or GSK-3β results in SNAIL degradation by the proteasome. This suggests that a good therapeutic target would be to induce the expression of GSK-3β to promote SNAIL degradation by the proteasome, which would in turn inhibit EMT. However, the inhibition of GSK-3β is also a viable therapeutic target [114]. As mentioned above, the design of therapeutic targets in cancer should be done with caution because many proteins can have dual functions as “suppressors” or “oncogenes” as in the case of GSK-3β and TGFβ whose biological effects may be different.

### SUMOylation

SUMOylation is another post-translational modification characterized by the reversible binding of Small Ubiquitin-like MOdifier (SUMO) to the target protein. The three-dimensional structure of SUMO is similar to ubiquitin [115, 116]. The SUMOylation of a target protein is mediated by a cascade of reactions involving an activating enzyme (e.g., SAE1/2), E2-conjugating enzyme (e.g., UBC9), and an E3 ligase. SUMO groups can be deconjugated by a group of Sentrin/SUMO-specific proteases (SENP) [116].

**Fig. 4:**
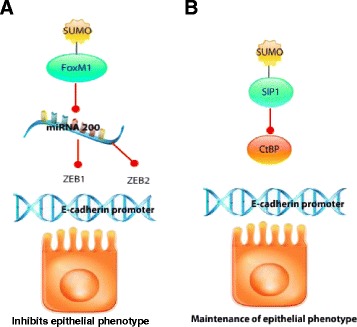
SUMOylation of SIP1 and FoxM1. (*Blue arrowhead* indicates sequential patterns.  indicates inhibition.) **a**. Sumoylation of FOXM1 represses the expression of miR-200b/c, which normally acts as a tumor suppressor and reduces the expression of transcription factors ZEB1 and ZEB2.As a consequence epithelial phenotype is inhibited. **b**. Sumoylation of SIP1 decreases its transcription, which in turn prevents recruitment of CtBP to E-cadherin promoter. As a consequence, CtBP cannot prevent E-cadherin expression, which results in the maintenance of the expression of epithelial genes

Forkhead box protein M1(FoxM1) is a transcription factor that belongs to a large family of forkhead box (Fox) transcription factors, which are characterized by the presence of a DNA-binding domain called the forkhead box or winged helix domain [117]. FoxM1 is expressed in proliferating cells and plays an important role in cell cycle progression stimulating the expression of genes involved in G_1_–S and G_2_–M progression [118]. It has been shown that FoxM1 is highly expressed in breast cancer [119]. FoxM1 can promote EMT through its direct binding at the SLUG promoter [120]. FoxM1 is subject to SUMOylation at lysine 463 and this posttranslational modification is required for the full repression of miR-200b/c in breast cancer cells [121]. Members of the miR-200 family act as tumor suppressive miRNAs, enhancing the expression of E-cadherin and suppressing the expression of ZEB1 and ZEB2. Thus, the overexpression of miR-200 results in a reduced expression of ZEB transcription factors and enhanced expression of epithelial makers [121–123]. In pancreatic cancer, FoxM1 is overexpressed and promotes EMT by the up-regulation of mesenchymal cell markers such as ZEB1, ZEB2, SLUG, and vimentin [117] (Fig. 4a).

The transcription factor activator protein 2C (TFAP2C) is very important in breast cancer biology, especially in luminal subtypes as it regulates genes including estrogen receptor-alpha (ERα) and HER2/c-erbB2. The expression of TFAP2C is critical for maintaining the luminal phenotype of breast cancer cells. The loss of TFAP2C in luminal breast cancer cell lines induces luminal to basal transition accompanied by an increase in the expression of mesenchymal markers (Vimentin and N-cadherin) and loss of E-cadherin. These results suggest that TFAP2C plays an important role in the regulation of luminal-specific genes [124]. TFAP2C undergoes SUMOylation that blocks its ability to induce the expression of luminal genes and helps it to maintain basal like features. The disruption of SUMOylation of TFAP2A induces a basal-to-luminal transition [125]. Although this mechanism is not directly related to EMT, it shows evidence of a transition to a more aggressive intrinsic subtype of breast cancer which as mentioned before, is characterized by high expression of mesenchymal genes.

Smad-Interacting Protein 1 (SIP1) is a member of the zfh-1 family and plays important functions during embryonic development. SIP1 has a binding motif for the corepressor C-terminal-binding protein (CtBP). SIP1 can induce EMT through the recruitment of CtBP to the *CDH1* promoter to repress its transcription. SIP1 is a target for SUMOylation mediated by the polycomb protein Pc2. This post- translational modification attenuates transcription of SIP1 and disrupts the recruitment of the corepressor CtBP. As a consequence, *CDH1* expression is maintained [126] (Fig. 4b). One of the mechanisms by which TGFβ regulates EMT is by the downregulation of the PIAS1 (protein inhibitor of activated STAT) which is a SUMO E3 ligase [127, 128]. It has been shown that the activation of PIAS1 suppresses the ability of TGFβ to activate matrix metalloproteinase 2 (MMP2) and invasive properties of breast cancer cells. In vivo, the disruption of the activity of PIAS1 in MDA-MB-231, enhance the development of bone metastasis after intracardiac injections of the cancer cells [127]. These results suggest that PIAS1 suppresses breast cancer metastasis through the inhibition of TGFβ.

SUMOylation plays an important role in the regulation of gene expression, genome instability, cellular functions, cellular senescence and stem cell reprogramming [129]. Furthermore, several investigators linked SUMO modification to other important diseases such as neurodegenerative disorders and heart diseases [130–133]. The addition of SUMO groups to transcription factors usually results in a decrease of gene expression of the target gene, which in turn results in the repression of E-cadherin and activation of mesenchymal genes.

### Glycosylation

The *O*-linked β-*N*-acetylglucosamine (*O*-GlcNAc) modification is a monosaccharide addition that occurs in nuclear and cytoplasmic proteins such as transcription factors, cytoskeletal proteins, nuclear pore proteins, oncogenes, and tumor suppressors specifically on serine (Ser) or threonine (Thr) residues. The uridine 5′-diphospho-N-acetylglucosamine (UDP-GlcNAc) is transferred to serine or threonine residues by the O-GlcNAc transferase (OGT) to produce the O-GlcNAc modification, while the removal of the modification is performed by an O-GlcNAcase [134, 135].

**Fig. 5:**
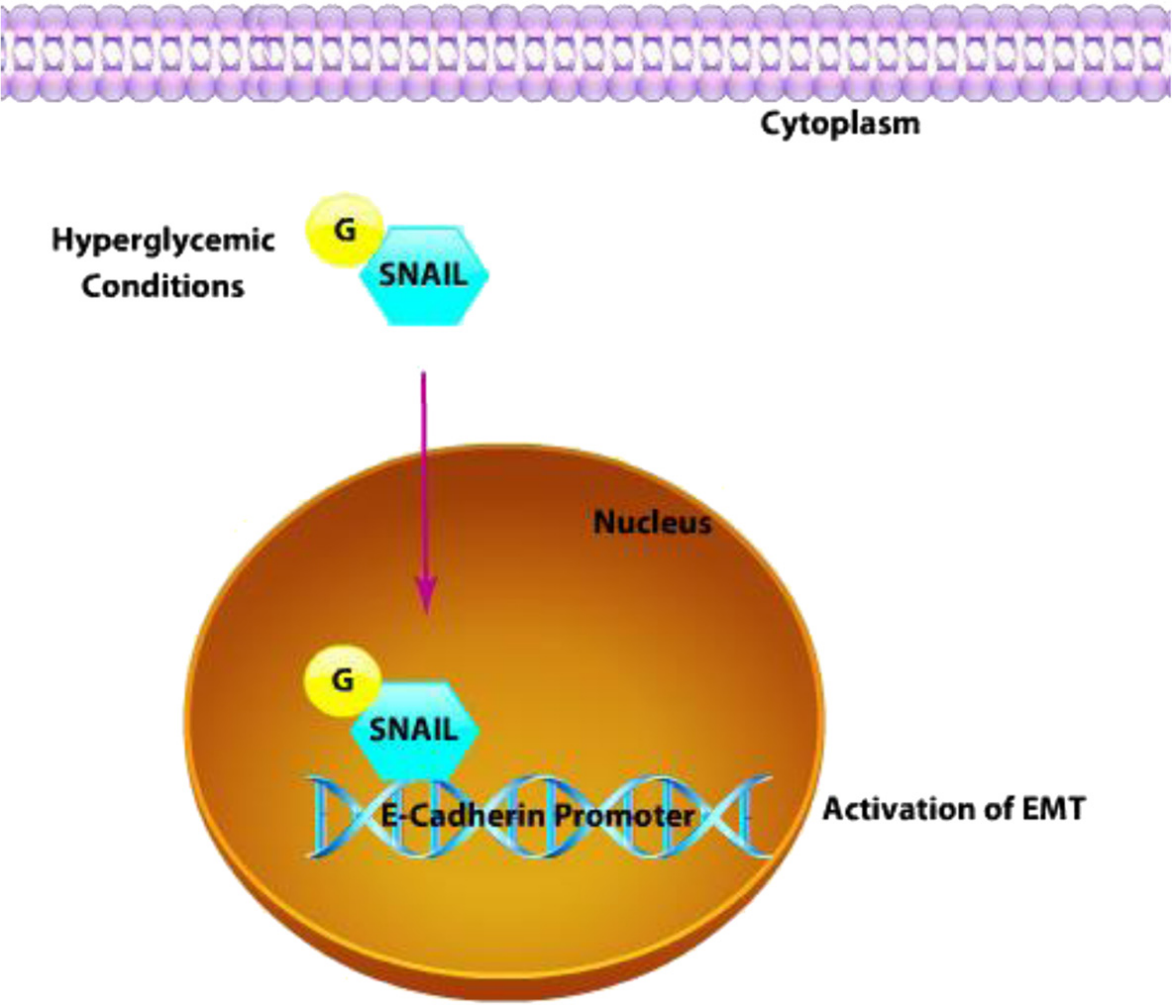
SNAIL Glycosylation. SNAIL is subject to O-linked glycosylation under hyperglycemic conditions. Consequently, the O-GlcNAc SNAIL promotes EMT by translocating it to the nucleus to bind to the E-cadherin promoter. (*Purple arrowhead* indicates translocation.)

In signal transduction cascades, the O-GlcNAcylation interplays with O-phosphorylation to regulate the function of several proteins [134, 136]. The inhibition of GSK3β results in the increase of O-GlcNAcylation of proteins including heat shock proteins, tubulin beta and vimentin; and at the same time decreases this modification on other proteins such as members of the hnRNP superfamily [136]. SNAIL is subject to O-GlcNAc at Ser 112 under hyperglycemic conditions. This modification leads to stabilization of SNAIL by inhibition of its O-phosphorylation, which is mediated by GSK3β. Consequently, the O-GlcNAc SNAIL promotes EMT [134] (Fig. 5). As discussed, EMT occurs due to the downregulation of epithelial markers and upregulation of mesenchymal markers such as vimentin, N-cadherin and fibronectin [137]. Oncofetal fibronectin (onfFN) is expressed in cancer or fetal cells/tissues, but not in normal adult cells/tissues and can be identified using mouse mAb FDC6. This antibody reacts with a specific O-glycosylated peptide sequence in IIICS domain of onfFN. It has been shown that the treatment with TGFβ in human prostate epithelial cell lines, induces the addition of O-glycan at a specific threonine at the type III homology connective segment (IIICS) domain of FN which is associated with the change in the morphology of the epithelial cells to fibroblast morphology, decrease of epithelial markers and an increase in the motility of these cells. The knockdown of this modification inhibits the TGF-β–induced up-regulation of onfFN and EMT process [138]. SNAIL is regulated by phosphorylation as well as glycosylation. Phosphorylation of SNAIL promotes its degradation. Oppositely, glycosylation of SNAIL stabilizes the protein under conditions of hypoglycemia. This raises one interesting question regarding the effect of glycosylation of SNAIL in normoglycemic conditions. It remains unclear what effect glycosylation of SNAIL has in cancer.

## Conclusion

Approximately 90 % of cancer mortalities occur in patients with tumors derived from epithelial tissues, and the primary cause of death in such cases results from dissemination of tumor cells to distant organs [139]. As such, understanding the cellular mechanisms contributing to metastasis is paramount in the effort to improve outcomes. EMT is a process in which tumor cells within the primary tumor lose their cell junctions and their epithelial morphology changes to fibroblastoid morphology. These changes allow the cells to invade the surrounding tissue of the primary tumor, intravasate into the bloodstream and lymphatic vessels as circulating tumor cells (CTC), and extravasate to distant sites where they may colonize distant organs as epithelial metastasis. Although EMT is a process that occurs under normal conditions such as wound healing and embryogenesis, the misappropriation of these pathways during tumor progression is an unpredictable and disastrous event with the simultaneous activation of different molecular cascades.

Many pharmacological approaches, including chemical inhibitors and monoclonal antibodies that target several proteins that regulate cancer progression have been devised and show promising results for the treatment of a variety of cancers. However, very little research has been done to target post translational modifications of proteins in cancers, and thus we believe that identifying inhibitors for post-translational modifications represents an underexplored area which may hold significant potential, and thus should be a high priority in the development of future cancer treatments. Furthermore, the identification of these post-transcriptional and post-translational modifications is important given that these changes could be identified in the primary tumor *before metastasis* occurs. Such knowledge would allow clinicians to better predict which patients have genotypes more likely to follow an aggressive clinical course prone to development of metastases. These patients could then be treated with different approaches from the onset of disease to reduce the risk of metastasis, and allow for better prognoses and ultimately, enhance survival.
